# Effect of season on the dynamics of cat sperm DNA fragmentation

**DOI:** 10.1186/s12917-023-03682-5

**Published:** 2023-08-08

**Authors:** Victoria Luño, Felisa Martínez, Andrea Muñoz, Lydia Gil

**Affiliations:** 1 Departament of Animal Pathology, Universidad de Zaragoza, Zaragoza, 50013 Spain; 2 Instituto Universitario de Investigación Mixto Agroalimentario de Aragón (IA2), Universidad de Zaragoza, Zaragoza, Spain

**Keywords:** Photoperiod, Tomcat, Spermatozoa, DNA fragmentation

## Abstract

**Background:**

Feline species undergo reproductive seasonality; thus, sperm characteristics, such as DNA integrity, can be affected by the photoperiod. This study was conducted to determine the effect of seasonal changes on sperm quality and on the dynamics of sperm DNA fragmentation. Epididymal spermatozoa were collected from 36 tomcats subjected to bilateral orchiectomy during breeding (BS) and non-breeding (NBS) seasons. Sperm samples were obtained by cutting the cauda epididymis and assessed for sperm motility, concentration, acrosome integrity, plasma membrane integrity and sperm morphology. Sperm DNA fragmentation was evaluated by the sperm chromatin dispersion test after 0, 6, and 24 h of incubation at 37 °C.

**Results:**

The total sperm motility and plasma membrane integrity values were greater during the BS, while the percentages of abnormal sperm and head defects were lesser (p < 0.05). No significant differences in DNA fragmentation were found between seasons after sperm collection. DNA damage was greater after 24 h of incubation at 37 °C in both seasons, although the percentage of spermatozoa with fragmented DNA was significantly lesser in the BS than in the NBS at 24 h (p < 0.05).

**Conclusions:**

The study suggests seasonal changes in some of the quality parameters of cat sperm. DNA fragmentation dynamics were affected by the time of incubation and reproductive season; therefore, this technique might be used as an additional tool to test the potential fertility of semen samples used in feline-assisted reproduction.

## Background

Cats undergo reproductive seasonality as an adaptive mechanism that allows for the concentration of parturitions in dates of greater food availability, thereby ensuring litter survival [1]. The breeding season changes according to geographic and environmental factors such as the temperature and number of daylight hours [2]. Queens are characterized by delimited seasonal reproductive activity, while in tomcats there are few studies related to this issue. Some authors describe an absence of breeding seasonality [3, 4]; however, other authors suggest seasonal modifications in sperm quality and functionality [5–7]. This variability of results seems to be related to the different collection methods, breeds or origins of animals used.

The information available about sperm quality in cats is relatively low in comparison to that in other domestic animals. In recent years, several studies have been performed on different feline semen collection, handling and conservation techniques due to its interest as an experimental model for the study of wild or endangered felines [7, 8]. An assessment of DNA status is not included in routine semen analysis, but this parameter could determine the fertilizing potential of an ejaculate. In tomcats, sperm DNA fragmentation is relatively low and does not increase during cold storage over time [9, 10]. In addition, there was no correlation between sperm morphology and the DNA fragmentation index [11].

The study of seasonal changes in sperm DNA integrity is limited in different species [12] and [13]. determined that the percentages of DNA damage were enhanced in December during the non-breeding season in stallions [14] and [15]. showed that ejaculates collected in late spring–summer showed relatively higher values of sperm DNA fragmentation [16]. reported that there was no seasonal change in sperm DNA fragmentation in ejaculates collected each month of the year. Similar contradictory results were obtained in boar ejaculates [17, 18]. In rams, chromatin showed more decondensation in summer, but no differences were observed between the breeding and non-breeding seasons [19].

Sperm DNA fragmentation is not a static process that allows each individual sperm to be assigned fixed data for the levels of damage; in contrast, it is a dynamic process. In several mammalian species, the rate of sperm DNA fragmentation increases when cells are exposed to an incubation temperature of 37 °C to mimic sperm transport in the female reproductive tract [20]. The measurement of DNA damage at multiple times could improve the knowledge of sperm characteristics at the time of fertilization.

The objective of the present study was to investigate the influence of season (breeding *versus* non-breeding) on tomcat sperm characteristics, especially on DNA integrity. In addition, the dynamics of DNA damage in sperm samples were analyzed over 24 h of incubation.

## Results

### Sperm characteristics during breeding and non-breeding seasons

The data for sperm motility parameters in both periods are summarised in Table 1. Compared to the NBS, the percentages of sperm total motility were higher during the BS (P = 0.026), although no differences were found in the progressive motility percentages. Additionally, compared to the NBS, the kinematics values in the BS were greater for VSL, VAP, LIN, STR and BCF (P < 0.05). In contrast, VCL, LIN and ALH were similar in both seasons. Sperm concentration and acrosome integrity values did not differ between seasons (Table 2). In contrast, sperm with intact plasma membrane were greater in the BS (P < 0.001). With regard to sperm morphology (Table 2), the number of morphologically normal spermatozoa was significantly higher during the BS than NBS (P < 0.001). The mean percentages of cytoplasmic droplets, sperm mid-piece and tail abnormalities were similar between the two groups. Head defects were greater during the NBS (P < 0.001), and they were the most commonly observed abnormalities.

**Table 1:** Effect of breeding (BS) and non-breeding seasons (NBS) on tomcats sperm motion characteristics (n = 18 in each group). Results are expressed as mean ± SD.

Sperm motility parameters	Season		*P-*value
	**BS**	**NBS**	
Total Motility (%)	59.20 ± 7.88^a^	53.23 ± 7.45^b^	0.026
Progressive Motility (%)	32.38 ± 5.90	31.61 ± 5.25	0.681
VCL (µm/s)	122.85 ± 4.98	117.19 ± 13.78	0.130
VSL (µm/s)	89.69 ± 4.69 ^a^	82.71 ± 5.74 ^b^	< 0.001
VAP (µm/s)	97.65 ± 7.54 ^a^	91.71 ± 7.23 ^b^	0.026
LIN (%)	83.54 ± 3.05 ^a^	77.81 ± 8.45 ^b^	0.011
STR (%)	78.18 ± 4.93 ^a^	73.78 ± 3.05	0.004
WOB (%)	88.71 ± 3.01	88.05 ± 2.96	0.523
ALH (µm)	3.92 ± 0.44	3.90 ± 0.22	0.875
BCF (Hz)	9.37 ± 0.38 ^a^	8.97 ± 0.48 ^b^	0.012

VCL: curvilinear velocity; VSL: straight-line velocity; VAP: average path velocity; STR: straightness; LIN: linearity of the curvilinear trajectory; STR: straightness; WOB: wobble; ALH: amplitude of lateral head displacement; BCF: beat cross frequency

a, b Different letters in the same motion characteristics indicate significant differences (p < 0.05)

**Table 2:** Effect of breeding (BS) and non-breeding seasons (NBS) on tomcats sperm quality parameters (n = 18 in each group). Results are expressed as mean ± SD.

Sperm parameters	Season		*P-*value
	**BS**	**NBS**	
Plasma membrane integrity (%)	60.33 ± 4.44 ^a^	54.78 ± 5.72 ^b^	0.003
Acrosomal integrity (%)	83.23 ± 2.66	81.44 ± 2.36	0.062
Concentration (10^6^ spz/ml)	77.35 ± 17.05	75.28 ± 10.95	0.779
Abnormal sperm (%)	22.05 ± 3.42 ^b^	27.06 ± 2.21 ^a^	< 0.001
Head defects (%)	4.95 ± 1.39 ^b^	10.22 ± 2.62 ^a^	< 0.001
Mid-pieces defects (%)	6.33 ± 1.53	5.28 ± 1.32	0.064
Tails defects (%)	5.28 ± 1.65	5.50 ± 1.57	0.727
Cytoplasmic droplets (%)	5.28 ± 1.84	6.34 ± 1.97	0.088

a, b Different letters in the same parameter indicates significant differences (p < 0.05)

### Effect of reproductive season on the DNA dynamics of cat sperm

**Fig. 1:**
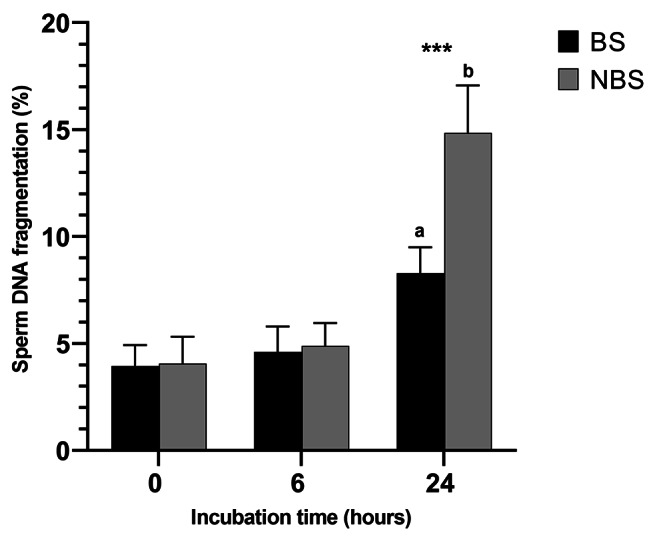
Dynamics of cat sperm DNA fragmentation between breeding and non-breeding seasons at each time of incubation at 37 °C (0, 6 and 24 h). a, b Different letters indicate significant differences between seasons (p < 0.05). Differences among different times of incubation (***; p < 0.05)

Figure 1 shows the averaged data of sperm DNA fragmentation dynamics after incubation at 37 °C for 24 h. No differences were observed between seasons at 0 and 6 h. However, the percentage of sperm with damaged DNA was significantly lesser in the BS than in the NBS at 24 h of incubation. In both seasons, the percentage of fragmented sperm DNA was significantly greater (P < 0.001) after 24 h at 37 °C. The interactions with respect to season and time are reported in Table 3. There were differences in both variables and in the interaction.

**Table 3:** Effect of season and incubation time at 37 °C on DNA fragmentation of 36 tomcats

Variable	DF	F	*P-*value
Season	1	75.69	< 0.001
Incubation time	2	320.201	< 0.001
Season x Incubation time	2	63.09	< 0.001

## Discussion

Feral cats have a reproductive season determined by photoperiod and melatonin secretion [21]. Therefore, similar to other seasonal species, males prepare for an intensification of their reproductive behaviour in specific periods of the year. There are changes in testosterone concentrations as a function of photoperiod, which can influence sperm production and quality [6, 22]. In the present study, the percentages of total sperm motility and viability showed higher values during the breeding season, in which high concentrations of plasmatic testosterone were observed [6]. These results are consistent with different studies performed with cats in several countries [22, 23]. However, [3] did not determine the changes in sperm quality parameters throughout the year. These differences may be due to collection methods, breeds or the origin of the animals. In our study, we utilized sperm samples from feral cats not from private owners, so feeding or domestic environmental factors did not influence in the results, as in some other studies.

Teratozoospermia is a frequent phenomenon in feline species [24]. Cats are considered teratozoospermic when the production of morphologically abnormal sperm in the ejaculate is > 60% [25], reaching values greater than 85% [24]. We determined that the percentages of abnormal sperm ranged from 22.05 to 27.06%, which are lower than those observed in other studies [5, 26]. The aetiology of teratozoospermia is unknown, occasionally appearing due to genetic, seasonal and nutritional factors or associated with abstinence periods and health status [6]. The age of the animal also seems to be related, as older cats showed higher percentages of abnormal sperm than younger cats [27]. In addition, [5] determined that the percentages of abnormal sperm were lower during the breeding season, especially head defects, as we observed in this study. Sperm abnormalities are related to a decrease in genetic variation and low concentrations of circulating testosterone [25], which occurs in the non-breeding season.

Sperm DNA integrity is essential to obtaining a successful pregnancy during natural mating as well as in assisted reproduction techniques. In the present work, DNA sperm damage was relatively low, independent of breeding season. These values are in agreement with those reported in the literature and obtained with different semen collection methods [11, 28, 29]. Since data on the effect of photoperiod on the DNA integrity of cat sperm have not yet been reported, we determined no differences in the DNA damage index during breeding and non-breeding seasons according to [16] in equine or [19] in ovine species. However, [14] and [15] reported that DNA damage of stallion sperm increased during late spring–summer and reached the lowest values in spring (the breeding season). They related DNA fragmentation to the high temperature values in summer months because of its effect on spermatogenesis. In contrast, [12] and [13] found that DNA fragmentation was enhanced in December during the non-breeding season. In a recent study, the majority of stallions had the least sperm DNA damage during the spring months, although a moderate proportion of the stallions showed the lowest value of sperm DNA damage during the winter months [30]. These results showed a large proportion of variability that was caused by the individuality of the stallions. On the other hand, boar sperm collected during an increasing photoperiod (especially in summer) have a relatively higher percentage of spermatozoa with fragmented DNA in comparison to those collected during a decreasing photoperiod (autumn) [18, 31]. The scrotum is not pendulous in boar, and spermatozoa tend to be more susceptible to temperature shock. In contrast, [17] reported that season, photoperiod or genetic line did not affect sperm DNA damage. Boars live in industrial artificial insemination studs with controlled environmental conditions; therefore, the influence of heat and season could be diminished.

Sperm DNA fragmentation could be measured at a single time (static assessment) or at multiple times at an incubation temperature of 37 °C that mimics sperm transport in the female reproductive tract (dynamic assessment) [20]. In this study, DNA damage was greater after 24 h of incubation at 37 °C in both seasons. Recently, [29] determined that the proportion of stallions with the lowest DNA fragmentation values in the breeding season increased from 45 to 60% when assessed after 0 and 24 h of incubation. These findings indicated that DNA dynamics evaluation is a more reliable technique for the evaluation of DNA damage because it can detect hidden changes in sperm DNA at the time when fertilisation occurs [20]. In addition, we only found significant seasonal variability with regard to sperm DNA damage after 24 h of incubation at 37 °C. In the reproductive season, a low amount of spermatozoa showed apoptotic-like features in semen [32]. The lack of continuous ejaculation during the non-breeding season may also increase reactive oxygen species production and apoptosis, leading to DNA having reduced resistance to heat stress. A dynamics assessment could have the potential to be an indicator of male fertility, and this technique might be used as an additional tool in the analysis of sperm quality prior to artificial insemination or cryopreservation [30].

Sperm chromatin is normally highly condensed and organized because histones are replaced by protamines during spermiogenesis. Protamine 1 (P1) is present in the sperm DNA of all mammals, whereas protamine 2 (P2) is found only in the sperm of primates, many rodents and a subset of other placental mammals [20]. The dynamic rate of DNA fragmentation is related to the P1/P2 ratio or to the number of cysteine groups in P1. The spermatozoa of those species without P2 showed lower DNA fragmentation percentages than those containing both P1 and P2 after temperature stress. The lack of P2 in the carnivore spermatozoa [33] might explain the resistance of DNA when incubated at 37 °C, which is different to that of stallion sperm [30]. Further studies are necessary to explain DNA fragmentation dynamics in relation to protamine quantity and male subfertility in tomcat sperm.

## Conclusion

In conclusion, most of the quality parameters of sperm were influenced by the reproductive season. Moreover, this study is the first to reveal differences in the sperm DNA fragmentation dynamics of cat spermatozoa between the breeding and non-breeding seasons. Measurement of DNA damage after 24 h at 37 °C could be used to select particular males as semen donors and to test the potential subfertility of the ejaculates utilized in assisted reproduction.

## Methods

### Reagents and media

All chemicals were obtained from Sigma‒Aldrich Chemical Company (Madrid, Spain) unless otherwise indicated.

### Animals and sperm recovery

The study was carried out in accordance with ARRIVE guidelines and Spanish Policy for Animal Protection RD 53/2013, which meets European Union Directive 2010/63/UE on animal protection. All experimental protocols were approved by the Ethical Committee of Animal Experimentation of the University of Zaragoza (nºPD32/20NE). Epididymal spermatozoa were collected from a total of 36 adult healthy feral cats (Felis catus) included in a program for breeding control by the Zaragoza City Council (Zaragoza, Spain). Sexual immature males (low testis weight, no sperm production) were discarded from the study. The animals were divided into two groups according to the date of castration. Group I cats were castrated during decreasing light (from October to November, n = 18), and Group II cats were castrated during increasing light (from April to May, n = 18). Once in the laboratory, the testes and epididymides were washed with a physiological saline solution (0.9% NaCl). The epididymides were then immediately dissected, and the spermatozoa were obtained by cutting the cauda epididymis in Tris solution (259 mM Trizma base, 80 mM citric acid and 69 mM fructose, 6.8 pH and an osmolality of 300/330 mOsm/kg) at 37 °C. The tissue was then washed for 10 min in an extender.

### Microscopic sperm evaluation

#### Computer-assisted sperm motility analysis

The motion parameters were determined using a computer-assisted sperm analysis (CASA) system (ISAS®; PROISER; Valencia, Spain). The samples were analysed at a concentration of 20 × 106 sperm/mL. The parameters evaluated were total motile spermatozoa (TM %), motile progressive spermatozoa (PM %), curvilinear velocity (VCL, µm/s), straight-line velocity (VSL, µm/s), average path velocity (VAP, µm/s), straightness (STR; ratio of VSL/VAP, %), linearity of the curvilinear trajectory (LIN; ratio of VSL/VCL, %), wobble (WOB; ratio of VAP/VCL, %), amplitude of lateral head (ALH, µm) and beat cross frequency (BCF; Hz). A 5 µL aliquot of each sperm sample was placed in a prewarmed Makler counting chamber. The setting parameters were 25 frames/s, in which spermatozoa had to be present in at least 15 frames to be counted. The sperm motility variable used in the statistical analysis was the overall percentage of motile spermatozoa (VCL > 20 μm/s). Images were obtained at 200× magnification using a contrast-phase microscope.

### Sperm concentration and morphology

To determine sperm concentration, an aliquot of the sample was diluted in formol saline, and spermatozoa were counted in a Bürker chamber. Sperm morphology was examined with Diff-Quick® (Microptic S.L., Barcelona, Spain) staining. At least 200 spermatozoa per slide were counted to determine the percentage of spermatozoa with abnormal morphology.

### Sperm plasma-membrane integrity

Sperm viability was evaluated using a LIVE/DEAD® sperm viability kit (Thermo Fisher Scientific, Hennigsdorf, Germany). Spermatozoa were mixed with SYBR-14 solution (10 µL/mL) and incubated at 37 °C for 10 min. The samples were then mixed with propidium iodide (PI) solution (2.4 mM) and incubated at 37 °C for 10 min. The proportion of live/dead sperm cells (200 sperm per sample) was measured at 400× magnification using a fluorescence microscope (Leica® DM2500 LED, l´Hospitalet del Llobregat, Spain).

### Acrosome status

The acrosomal membrane integrity was assessed by fluorescein isothiocyanate conjugated with peanut agglutinin (FITC-PNA) and propidium iodide (PI) staining. Spermatozoa were mixed with FITC-PNA solution (200 µg/mL) and PI solution (500 µg/mL), kept at 38 °C for 5 min, and finally fixed in paraformaldehyde (4% (v/v) in saline solution. At least 200 spermatozoa were examined under a fluorescence phase-contrast microscope. The data corresponding to viable spermatozoa (intact plasma and acrosomal membranes; PNA−/PI−) were determined using a fluorescence microscope.

### DNA integrity

**Fig. 2:**
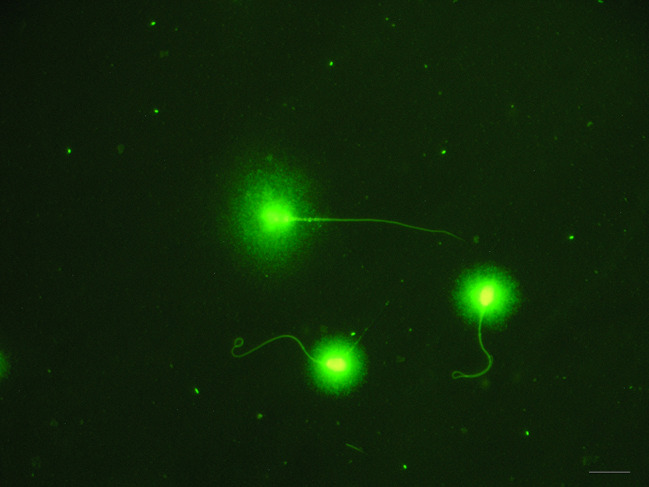
Cat sperm processed with Halomax kit®. Those with a small halo have normal status of DNA and the spermatozoon with a large halo contains fragmented DNA. Scale bar represents 10 μm.

Sperm DNA fragmentation was evaluated by a sperm chromatin dispersion test specifically designed for cat spermatozoa (Halomax®, Halotech DNA SL, Madrid, Spain). DNA fragmentation analysis in all groups was performed following the instructions of the manufacturer. In brief, the lysis solution was placed at room temperature (22 °C). Then, an Eppendorf tube containing agarose was placed in a 95–100 °C water bath for five minutes before being transferred to a 37 °C water bath for five minutes. Additionally, 25 µl of each diluted sperm sample was added to an empty Eppendorf tube, and 50 ml of liquefied agarose was then transferred into the tube and gently mixed. The temperature of the tubes was maintained at 37 °C. A 2 µl drop of the cell suspension was placed onto marked wells, and each drop was covered with a 24 × 24 mm glass coverslip. The slides were held in a horizontal position throughout the entire process. The slides were placed on a cold surface precooled to 4 °C in a refrigerator to solidify the agarose. After 5 min, the slides were withdrawn from the refrigerator, and the coverslips were gently removed. Then, the slides were fully immersed horizontally in 10 ml of lysis solution for five minutes. Subsequently, the preparation was introduced into a bath of distilled water for 5 min and then dehydrated by immersion in 2 successive baths of ethanol, 70% and then 100%, for 2 min each. Finally, the slides were allowed to air-dry before staining. All slides were stained using a commercial kit for green fluorescence staining (Fluogreen, Halotech DNA SL, Madrid, Spain). Briefly, 2 µl of green fluorochrome and mounting medium (1:1; vol/vol) was placed into the well of the slide for the fluorescent staining of sperm chromatin. The samples were evaluated using fluorescence microscopy (Olympus BX-40 Olympus U-RFL-T, Tokyo, Japan) at a 400× magnification and a minimum of 500 spermatozoa were counted per semen sample. Sperm showing a small and compacted halo around a compacted nuclear core contained intact DNA, and sperm that displayed a large and spotty halo around the nuclear core corresponded to sperm with damaged DNA (Fig. 2). The sperm DNA fragmentation index was calculated as the percentage of sperm with fragmented DNA over the total number of sperm counted.

### Experimental design

Sperm quality variables (sperm motility, concentration, viability, acrosome status, morphology and DNA integrity) were assessed at the time of collection with respect to the breeding (BS) and non-breeding seasons (NBS). The effect of season on sperm DNA dynamics was determined by the sperm chromatin dispersion test after sperm sample incubation at 37 °C in a water bath for 0, 6 and 24 h.

### Statistical analysis

The statistical analysis was performed using SPSS version 22.0 for Windows (Chicago, IL, USA). Kolmogórov–Smirnov tests were used to verify the normality of the values. Differences in sperm quality during breeding and non-breeding seasons were examined by one-way ANOVA. The dynamics of DNA sperm damage were analysed using a linear mixed model, including the fixed effect of season and time. When analysis of variance showed a significant effect, values were compared using the least-significant-difference pairwise multiple-comparisons post hoc test (Tukey HSD test). The data are expressed as the mean value ± standard deviation (SD). Differences were considered statistically significant at p < 0.05.

## Data Availability

The datasets used and/or analyzed during the current study are available from the corresponding author on reasonable request

## References

[1] Leyva H, Madley T, Stabenfeldt GH (1989). Effect of light manipulation on ovarian activity and melatonin and prolactin secretion in the domestic cat. J Reprod Fertil Suppl.

[2] Chemineau P, Guillaume D, Migaud M, Thiery JC, Pellicer-Rubio MT, Malpaux B (2008). Seasonality of reproduction in mammals: intimate regulatory mechanisms and practical implications. Reprod Domest Anim.

[3] Spindler RE, Wildt DE (1999). Circannual variations in intraovarian oocyte but not epididymal sperm quality in the domestic cat. Biol Reprod.

[4] Franca LR, Godinho CL (2003). Testis morphometry, seminiferous epithelium cycle length, and daily sperm production in domestic cats (Felis catus). Biol Reprod.

[5] Axner E, Linde-Fosberg C (2007). Sperm morphology in the domestic cat and its relation with fertility: a retrospective study. Reprod Domest Anim.

[6] Blottner S, Jewgenow K (2007). Moderate seasonality in testis function of domestic cat. Reprod Domest Anim.

[7] Stornelli MA (2007). Evaluación de semen en gato doméstico: análisis de rutina y metodologías especiales. Rev Bras Reprod Anim.

[8] Sowińska N (2021). The domestic cat as a research model in the assisted reproduction procedures of wild felids. Postepy Biochem.

[9] Filliers M, Rijsselaere T, De Causmaecker V, Bossaert P, Dewulf J, Pope CE, Van Soom A (2008). Computerassisted semen analysis of fresh epididymal cat spermatozoa and the impact of cooled storage (4°C) on sperm quality. Theriogenology.

[10] Buarpung S, Tharasanit T, Comizzoli P, Techakumphu M (2012). Effects of cold storage on plasma membrane, DNA integrity and fertilizing ability of feline testicular spermatozoa. Anim Reprod Sci.

[11] Vernocchi V, Morselli MG, Consiglio AL, Faustini M, Luvoni GC (2014). DNA fragmentation and sperm head morphometry in cat epididymal spermatozoa. Theriogenology.

[12] Blottner S, Warnke C, Tuchscherer A, Heinen V, Torner H (2001). Morphological and functional changes of stallion spermatozoa after cryopreservation during breeding and non-breeding season. Anim Reprod Sci.

[13] Morte MI, Rodrigues AM, Soares D, Rodrigues AS, Gamboa S, Ramalho-Santos J (2008). The quantification of lipid and protein oxidation in stallion spermatozoa and seminal plasma: seasonal distinctions and correlations with DNA strand breaks, classical seminal parameters and stallion fertility. Anim Reprod Sci.

[14] Wach-Gygax L, Burger D, Malama E, Bollwein H, Fleisch A, Jeannerat E (2017). Seasonal changes of DNA fragmentation and quality of raw and cold-stored stallion spermatozoa. Theriogenology.

[15] Crespo F, Quiñones-Pérez C, Ortiz I, Diaz-Jimenez M, Consuegra C, Pereira B, Dorado J, Hidalgo M (2020). Seasonal variations in sperm DNA fragmentation and pregnancy rates obtained after artificial insemination with cooled-stored stallion sperm throughout the breeding season (spring and summer). Theriogenology.

[16] Janett F, Burger D, Bollwein H (2014). Annual variation of DNA fragmentation assessed by SCSA in equine sperm. J Equine Vet Sci.

[17] Petrocelli H, Batista C, Gosálvez J (2015). Seasonal variation in sperm characteristics of boars in southern Uruguay. Rev Bras Zootec.

[18] Ausejo R, Martínez JM, Soler-Llorens P, Bolarín A, Tejedor T, Falceto MV (2021). Seasonal changes of nuclear DNA fragmentation in Boar Spermatozoa in Spain. Anim (Basel).

[19] García-Macías V, Martínez-Pastor F, Alvarez M, Borragan S, Chamorro CA, Soler AJ, Anel L, de Paz P (2006). Seasonal changes in sperm chromatin condensation in ram (Ovis aries), Iberian red deer (Cervus elaphus hispanicus), and brown bear (Ursus arctos). J Androl.

[20] Gosálvez J, Lopez-Fernandez C, Fernandez JL, Gouraud A, Holt WV (2007). Relationships between the dynamics of iatrogenic DNA damage and genomic design in mammalian spermatozoa from eleven species. Mol Reprod Devel.

[21] Robinson R, Cox HW (1970). Reproductive performance in a cat colony over a 10-year period. Lab Anim.

[22] Tsutsui T, Onodera F, Oba H, Mizutani T, Hori T (2009). Plasma hormone levels and semen quality in male cats during non-breeding and breeding seasons. Reprod Domest Anim.

[23] Nuñez-Favre R, Bonaura M, Tittarelli C, Mansilla-Hermann D, de la Sota R, Stornelli M (2012). Effect of natural photoperiod on epididymal sperm quality and testosterone serum concentration in domestic cat (Felis silvestris catus). Reprod Domest Anim.

[24] Pukazhenthi BS, Wildt DE, Howard JG (2001). The phenomenon and significance of teratospermia in felids. J Reprod Fertil.

[25] Howard JG, Brown JL, Bush M, Wildt DE (1990). Teratospermic and normospermic domestic cats: ejaculate traits, pituitary-gonadal hormones, and improvement of spermatozoal motility and morphology after swim-up processing. J Androl.

[26] Axnér E, Linde-Forsberg C, Einarsson S (1999). Morphology and motility of spermatozoa from different regions of the epididymal duct in the domestic cat. Theriogenology.

[27] Jewgenow K, Neubauer K, Blottner S, Schön J, Wildt DE, Pukazhenthi BS (2009). Reduced germ cell apoptosis during spermatogenesis in the teratospermic domestic cat. J Androl.

[28] Mota PC, Ramalho-Santos J (2006). Comparison between different markers for sperm quality in the cat: Diff-Quik as a simple optical technique to assess changes in the DNA of feline epididymal sperm. Theriogenology.

[29] Thuwanut P, Chatdarong K, Techakumphu M, Axnér E (2008). The effect of antioxidants on motility, viability, acrosome integrity and DNA integrity of frozen-thawed epididymal cat spermatozoa. Theriogenology.

[30] Crespo F, Wilson R, Díaz-Jimenez M, Consuegra C, Dorado J, Barrado BG, Gosálvez J, Smit RL, Hidalgo M, Johnston S (2020). Effect of season on individual stallion semen characteristics. Anim Reprod Sci.

[31] Zasiadczyk L, Fraser L, Kordan W, Wasilewska K (2015). Individual and seasonal variations in the quality of fractionated boar ejaculates. Theriogenology.

[32] Mendoza N, Casao A, Domingo J, Quintín F, Laviña A, Fantova E, Cebrián-Pérez J, Muiño-Blanco T, Pérez-Pe R (2021). Influence of non-conventional sperm quality parameters on Field Fertility in Ovine. Front Vet Sci.

[33] Lee CH, Cho YH (1999). Aspects of mammalian spermatogenesis: electrophoretical analysis of protamines in mammalian species. Mol Cells.

